# Prognostic significance of steroid response in pediatric acute lymphoblastic leukemia: The CCCG-ALL-2015 study

**DOI:** 10.3389/fonc.2022.1062065

**Published:** 2022-12-23

**Authors:** Jinhua Chu, Huaju Cai, Jiaoyang Cai, Xinni Bian, Yumei Cheng, Xianmin Guan, Xiaoqian Chen, Hua Jiang, Xiaowen Zhai, Yongjun Fang, Lei Zhang, Xin Tian, Fen Zhou, Yaqin Wang, Lingzhen Wang, Hong Li, Leung Wing Kwan Alex, Minghua Yang, Hanfang Yang, Aijun Zhan, Ningling Wang, Shaoyan Hu

**Affiliations:** ^1^Department of Hematology/Oncology, Pediatrics, the Second Hospital of Anhui Medical University, Hefei, China; ^2^Department of Hematology/Oncology, Shanghai Children’s Medical Center, Shanghai Jiaotong University of School of Medicine, Shanghai, China; ^3^Department of Hematology/Oncology, Children’s Hospital of Soochow University, Suzhou, China; ^4^Department of Pediatrics, Institute of Hematology and Blood Disease Hospital, Chinese Academy of Medical Sciences and Peking Union Medical College, Tianjin, China; ^5^Department of Hematology/Oncology, Children’s Hospital of Chongqing Medical University, Chongqing, China; ^6^Hematology/Oncology, West China Second Hospital of Sichuan University, Chengdu, China; ^7^Department of Hematology/Oncology, Guangzhou Women and Children Health Care Center, Guangzhou, China; ^8^Department of Hematology/Oncology, Children’s Hospital of Fudan University, Shanghai, China; ^9^Department of Hematology/Oncology, Nanjing Children’s Hospital Affiliated to Nanjing Medical University, Nanjing, China; ^10^Department of Pediatrics, Nanfang Hospital, Southern Medical University, Guangzhou, China; ^11^Department of Hematology/Oncology, Kunming Children’s Hospital, Kunming, China; ^12^Department of Pediatrics, Huazhong University of Science and Technology Tongji Medical College Union Hospital, Wuhan, China; ^13^Department of Pediatrics, Huazhong University of Science and Technology Tongji Medical College Tongji Hospital, Wuhan, China; ^14^Department of Pediatrics, Affiliated Hospital of Qingdao University, Qingdao, China; ^15^Department of Hematology Oncology, Children’s Hospital Affiliated to Shanghai Jiaotong University, Shanghai, China; ^16^Department of Pediatrics, The Chinese University of Hong Kong, Hong Kong Children’s Hospital, Hong Kong, China; ^17^Department of Pediatrics, Xiangya Hospital Central South University, Changsha, Hunan, China; ^18^Department of Hematology/Oncology, Northwest Women’s and Children’s Hospital, Xi’an, China; ^19^Department of Pediatrics, Qilu Hospital of Shandong University, Jinan, China

**Keywords:** acute lymphocytic leukemia, steroid response, children, overall survival, event-free survival

## Abstract

**Introduction:**

Whether steroid response is an independent risk factor for acute lymphoblastic leukemia (ALL) is controversial. This study aimed to investigate the relationship between response to dexamethasone and prognosis in children with ALL.

**Methods:**

We analyzed the data of 5,161 children with ALL who received treatment in accordance with the Chinese Children’s Cancer Group ALL-2015 protocol between January 1, 2015, and December 31, 2018, in China. All patients received dexamethasone for 4 days as upfront window therapy. Based on the peripheral lymphoblast count on day 5, these patients were classified into the dexamethasone good response (DGR) and dexamethasone poor response (DPR) groups. A peripheral lymphoblast count ≥1× 10^9^/L indicated poor response to dexamethasone.

**Results:**

The age, white blood cell counts, prevalence of the *BCR/ABL1* and *TCF3/PBX1* fusion genes, and rates of recurrence in the central nervous system were higher in the DPR than in the DGR group (*P*<0.001). Compared to the DPR group, the DGR group had a lower recurrence rate (18.6% vs. 11%) and higher 6-year event-free survival (73% vs. 83%) and overall survival (86% vs. 92%) rates; nevertheless, subgroup analysis only showed significant difference in the intermediate-risk group (*P*<0.001).

**Discussion:**

Response to dexamethasone was associated with an early treatment response in our study. In the intermediate-risk group, dexamethasone response added a prognostic value in addition to minimal residual disease, which may direct early intervention to reduce the relapse rate.

## Introduction

Acute lymphocytic leukemia (ALL) is the most common malignancy among children and accounts for 25% of all childhood malignancies (1). Over the past 20 years, the event-free survival (EFS) and overall survival (OS) rates among pediatric patients with ALL have exceeded 80% and 90%, respectively (2–6). Currently, the known prognostic factors of ALL include the patient’s age, white blood cell (WBC) count at initial diagnosis, immunophenotype, extramedullary leukemia status,

tumor cytogenetic and biological characteristics, and treatment response (7). In the 1980s, the Berlin–Frankfurt–Munster (BFM) study group found that patients with a poor prednisone response had a poor prognosis (8). The D8 prednisone response was also included as an independent prognostic factor in the risk stratification criteria of the following: 1) the “Associazione Italiana di Ematologia Oncologia Pediatrica and the Berlin-Frankfurt-Münster Acute Lymphoblastic Leukemia” study in 2000, 2) the 2005 ALL program of the Shanghai Children’s Medical Center (SCMC-ALL-2005) protocol, and 3) the Chinese Children’s Leukemia Group (CCLG)-ALL-2008 protocol (9, 10). Good and poor responses were defined by the presence of <1 × 109/L or ≥1 × 109/L blasts in the blood, respectively, after a 7-day prednisone prophase (11).

In the CCLG-ALL-2008 trial, patients with a poor hormone response were included in the high-risk (HR) group. However, the EFS rate, recurrence rate, and time-to-recurrence did not differ significantly between children in the HR group with DGR and DPR. This indicated that the prednisone response had a limited prognostic value among patients in the HR group (10). Multivariate Cox regression analysis of the early treatment response and prognosis in the SCMC-ALL-2005 protocol indicated that the response to prednisone treatment had no significant effects on the prognosis (9). Thus, all of these studies have indicated that the prednisone response affects the prognosis only in some patients.

The protocol for the Chinese Children’s Cancer Group ALL-2015 (CCCG-ALL-2015) trial has been completed since 2 years and produced several achievements within a large cohort of children with ALL (12–17). However, the role of dexamethasone on the prognosis has not been evaluated. The present study summarized the data of 5,161 children who were newly diagnosed with ALL and were assessed after a 4-day treatment with dexamethasone. The objectives of the current study were to determine whether there exists a correlation between response to dexamethasone and prognosis.

## Materials and methods

### Ethics and consent

This study was approved by the Central Institutional Review Board (Approval number: SCMCIRB-K2014060) and was performed in accordance with the Declaration of Helsinki and its later amendments. Written informed consent was obtained from parents, guardians, or patients.

### CCCG-ALL-2015 trial and study design

The enrolled participants were children who were newly diagnosed with ALL between January 1, 2015, and December 31, 2018. Compared with prednisone, dexamethasone has a wider tissue distribution, better blood–brain barrier penetration, and a stronger anti-leukemia effect. The participants were exposed to dexamethasone (6 mg/m2 per day) during a 4-day treatment window. The immature lymphocyte count in the peripheral blood was assessed on day (d) 5. Early treatment responses were assessed using cytological findings of the bone marrow and the minimal residual disease (MRD) on d19 and d46 of induction therapy as the indicator for the risk stratification criteria. However, responses to dexamethasone in the treatment window were not considered early treatment responses or risk stratification criteria.

The study protocol included a central review of the MRD and major adverse events every 6 months, periodic internal and on-site monitoring, and external auditing to ensure protocol compliance and appropriate data management.

### Participants

We enrolled patients who were aged 0–18 years, were newly diagnosed with ALL, and had at least completed induction chemotherapy. Patients with secondary malignancies or primary immunodeficiencies were not eligible for enrolment; only those with a history of steroid treatment for <3 days were allowed. A total of 5,161 children were eligible, including 3,067 boys and 2,094 girls (median age, 4.6 years; range, 42 days to 17 years). Among these, 4,698 and 463 patients had B-cell ALL (B-ALL) and T-cell ALL (T-ALL), respectively. Furthermore, a total of 2,650, 2,401, and 110 patients were classified as low-risk (LR), intermediate-risk (IR), and HR, respectively.

### Dexamethasone response in the pretreatment phase

Traditionally, prednisone is used for window therapy in childhood ALL; yet, dexamethasone may have higher anti-leukemic potency, leading to fewer relapses and improved survival (10). In this study, the children received 6 mg/m2 of dexamethasone per day for 4 days (d1–d4). The peripheral lymphoblast count was assessed on d5. If the patient had a WBC count of ≥50 × 109/L on d0, 3 mg/m2 of dexamethasone was administered as additional dose. If the peripheral lymphoblast count on d5 was <1 × 109/L, the dexamethasone response was considered good, and if the count was ≥1 × 109/L, the dexamethasone response was considered poor.

### Procedures

All patients received dexamethasone for 4–5 days as upfront window therapy, followed by remission induction. Specific schemes and risk stratification are shown in previously published articles (11, 14–16).

### Follow-up

The data were collected from our research center by designated personnel using an information collection form predesigned based on the protocol and entered into the pediatric ALL database within one month of ALL diagnosis. All patients were followed-up, and a follow-up observation form was completed at each follow-up examination. The follow-up information in the database was updated every 6 months. The last follow-up examination for this study was on July 31, 2021. A total of 62 patients were lost to follow-up before the study endpoint (event or death) was reached. The loss-to-follow-up rate was 1.20%. OS was defined as the time from the start of group-based treatment to death or last follow-up examination. EFS was defined as the time from the initial diagnosis to the occurrence of the first event (recurrence, death, or development of a second tumor) or the last follow-up examination. Withdrawal was defined as failure of patients to complete the treatment without the aggravation or recurrence of leukemia because the parents of the patients voluntarily refused treatment. Loss to follow-up was defined as failure to obtain follow-up information from patients who completed all treatments. This protocol was initiated on January 1, 2015 and ended on July 31, 2021. The median follow-up duration was 52.5 months (0.3–79.7 months).

### Statistical methods

SPSS 20.0 software (IBM Corp., Armonk, NY, USA) was used to plot the Kaplan–Meier survival curves for the DGR and DPR groups. Survival curves for the two groups were compared using log-rank tests. Measurement data were compared using the independent sample rank-sum test. Count data are expressed as frequencies and percentages, and comparisons between groups were performed using the chi-square test or Fisher’s exact probability test. Joint effects and independent factors of poor treatment outcomes were analyzed using logistic regression. Statistical significance was set at *P*<0.05.

## Results

**Figure 1:**
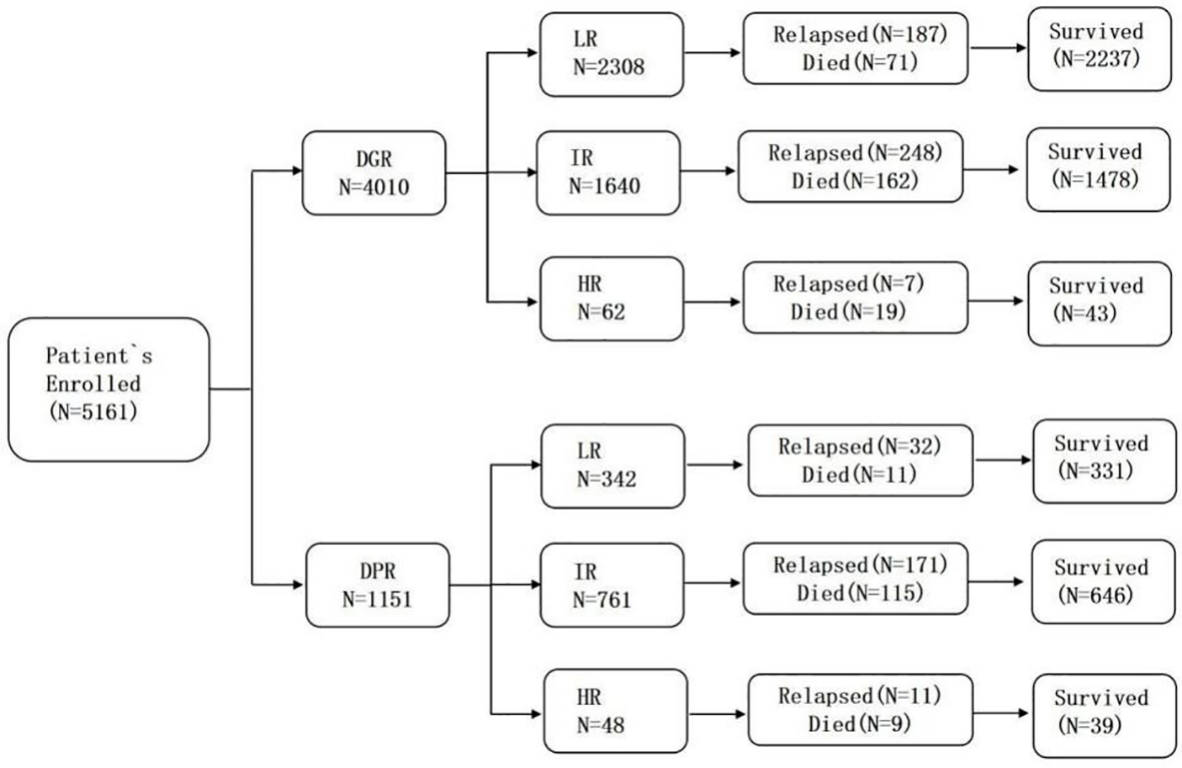
Flow chart of distribution.

In total, 5,161 children were assessed after they completed window treatment with dexamethasone. There were 4,010 patients in the DGR group (77.7%) and 1,151 in the DPR group (22.3%). The biological characteristics of the two groups are presented in **Table 1** and **Figure 1**. Testicular involvement, mixed-lineage leukemia (MLL) rearrangement, and C-myc break-apart at the time of initial diagnosis were not significantly different between the two groups (*P*>0.05). Univariate analysis indicated a significant difference in age distribution between the two groups (*P*=0.013). Further analysis showed that the proportion of patients aged ≥10 years was significantly higher in the DPR than in the DGR group (*P*=0.01, a=0.017). In addition, the WBC count, sex composition, immunophenotype, risk, central nervous system (CNS) involvement, and genetic characteristics at the time of initial diagnosis were significantly different between the two groups. Compared to the DGR group, the DPR group included more patients with high WBC counts at the time of initial diagnosis (43.52% vs. 15%); a higher male-to-female ratio (*P*=0.008); a higher proportion of patients with T-lineage All (16.1% vs. 6.9%); a lower proportion of patients at LR (29.7% vs. 57.6%); a higher proportion of patients with *BCR/ABL1* (7.04% vs. 3.34%), *TCF3/PBX1* (7.65% vs. 4.44%), and *PDGFRB* fusion genes (0.7% vs. 0.17%); and a lower proportion of patients with *EVT6/RUNX1* fusion genes (11.64%vs. 21.05%). Karyotypes between the two groups were significantly different (*P<*0.001), and the proportion of abnormal karyotypes was significantly higher in the DPR than in the DGR group (*P<*0.05, a=0.017). According to risk and sex analysis, male individuals were significantly predominant among intermediate-to-high-risk children than among children at LR (*P*=0.016).

**Table 1:** Biological features of children with acute lymphoblastic leukemia according to dexamethasone response.

Clinical characteristics	DGR	DPR	*P*-value
Age (year)			0.013
<1	50 (1.3%)	21 (1.8%)	
1–9	3453 (86.1%)	952 (82.7%)	
≥10	507 (12.6%)	178 (15.5%)	
Sex			0.008
Male	2336 (58.3%)	721 (62.6%)	
Female	1674 (41.7%)	430 (37.4%)	
Initial white blood cell grades, ×10^3^/uL			<0.001
≥100	289 (7.2%)	277 (24%)	
≥50	313 (7.8%)	224 (19.5%)	
<50	3408 (85%)	650 (56.5%)	
Central nervous system invasion	29 (0.7%)	31 (2.7%)	<0.001
Testicular aggression in male children	11 (0.27%)	6 (0.52%)	0.239
Initial risk stratification			<0.001
LR	2308 (57.6%)	342 (29.7%)	
IR	1640 (40.9%)	761 (66.1%)	
HR	62 (1.5%)	48 (4.2%)	
Immunophenotype			<0.001
B-ALL	3732 (93.1%)	966 (83.9%)	
T-ALL	278 (6.9%)	185 (16.1%)	
Karyotype analysis			<0.001
Normal	2577	731	
≥50	532	156	
Others abnormal karyotype	213	109	
Genetics			
t(12,21); EVT6/RUNX1	844 (21.05%)	134 (11.64%)	<0.001
t(9,22); BCR/ABL1	134 (3.34%)	81 (7.04%)	<0.001
t(1,19); TCF3/PBX1	178 (4.44%)	88 (7.65%)	<0.001
t(v;11q23); MLL	106 (2.64%)	53 (4.61%)	1
C-myc breakage	9 (0.22%)	2 (0.17%)	1
PDGFRB	7 (0.17%)	8 (0.70%)	0.008

DGR, dexamethasone good response; DPR, dexamethasone poor response; HR, high-risk; IR, intermediate-risk; LR, low-risk; WBC, white blood cell count.

Univariate analysis included factors, such as age, sex, initial WBC count, risk, and immunophenotype. There were obvious differences in the response to dexamethasone treatment. Multivariate analysis seen in **Table 2** indicated that the WBC count (odds ratio, 3.207; 95% confidence interval, 2.714–3.791; *P*<0.001), age (*P*=0.007), and risk (*P*<0.001) were significantly different between the two groups, but there were no significant differences in sex (*P*=0.141) or immunophenotype (*P*=0.144).

**Table 2:** Multivariate analysis of the clinical characteristics associated with dexamethasone response.

Clinical characteristic	Group	OR	95% CI		*P*-value
			Minimum	Maximum	
Age (year)					
<1		1.501	0.859	2.622	0.154
≥1 and <10		0.756	0.617	0.927	0.007
≥10		-	-	-	-
Sex					
Male		0.898	0.778	1.037	0.141
Female					
Initial white blood grades					
<50		3.207	2.714	3.791	<0.001
≥50		-	-	-	-
Initial risk stratification					
LR		3.758	2.468	5.723	<0.001
IR		1.787	1.193	2.677	0.005
HR		-	-	-	-
Immunophenotype					
B-ALL		1.18	0.945	1.474	0.144
T-ALL		-	-	-	-

LR, low risk; HR, high risk, IR, intermediate risk; B-ALL, B-cell acute lymphoblastic leukemia, T-ALL, T-cell lymphoblastic leukemia; CI, confidence interval.

### Early treatment response in the DGR and DPR groups

**Table 3** provides data on the relationship between dexamethasone sensitivity and early treatment response. Bone marrow MRD on d19 and d46 of induction chemotherapy was significantly different between the two groups (*P<*0.001). On d19, the bone marrow MRD (D_19_MRD) in the DPR group was significantly higher than that in the DGR group, while the proportion of patients with MRD ≥10-2 was 32.9% in the DPR group and 14.1% in the DGR group. On day 46, the proportion of patients with a bone marrow MRD (D_46_MRD) ≥10^-4^ was 18.9% in the DPR group and 11.2% in the DGR group.

**Table 3:** Minimal residual disease for children with acute lymphoblastic leukemia according to dexamethasone response.

Group	D_19_MRD				D_46_MRD		
	≥10^-2^	10^-3^–10^-2^	10^-4^–10^-3^	<10^-4^	≥10^-2^	10^-4^–10^-2^	<10^-4^
DGR(n)	565 14.1%	737 18.4%	645 16.1%	2063 51.4%	60 1.5%	388 9.7%	3562 88.8%
DPR(n)	374 32.9%	234 22.3%	119 10.3%	424 34.5%	44 3.8%	173 15.1%	934 81.1%
*P*-value	<0.001				<0.001		

DGR, dexamethasone good response; DPR, dexamethasone poor response; D_19_MRD, minimal residual disease on day 19 of induction; D_46_MRD, minimal residual disease on day 49 of induction.

### Relationship between early treatment response and relapse in the two groups

As reported in **Table 4**, in the DGR group, LR children with D_19_MRD ≥10^-3^ or D_46_MRD ≥10^-4^ had a significantly higher recurrence rate than those in the other risk subgroups (*P*<0.05). IR children with D_19_MRD ≥10^-4^ or D_46_MRD ≥10^-4^ had a significantly higher recurrence rate than those in the other risk subgroups (*P*<0.05). In addition, intermediate-risk children with D_19_MRD ≥10^-2^ had a recurrence rate similar to that of children with 10^-4^ ≤D_19_MRD <10^-3^ and lower than that of children with 10^-3^ ≤D_19_MRD<10^-2^, a finding that might be associated with the extra course of CAT+ consolidation therapy (vincristine, VCR, 1.5 mg/m^2^ intravenously [IV] on d1, d8; Peg-Asp, 2000 U/m^2^ intramuscular on d1; cyclophosphamide, CTX, 1000 mg/m^2^ IV on d1; cytarabine, Ara-C, 50 mg/m^2^ IH q12h d1–7; 6-mp, 60 mg/m^2^ po on d1–7 in the former). Among the 62 children at HR, D_19_MRD and D_46_MRD were not significantly correlated with recurrence (*P*=1). In the DPR group, D_19_MRD and D_46_MRD were not significantly correlated with recurrence in patients at LR or HR (*P*>0.05). However, patients at IR with a D_19_MRD <10^-4^ or D_46_MRD <10^-4^ had a significantly lower recurrence rate than those in the other risk subgroups (*P*=0.013 and *P*<0.05, respectively). Patients at IR with D_19_MRD ≥10^-2^ had the highest recurrence rate compared to those at other risk subgroups. Children at IR with D_19_MRD <10^-4^ or D_46_MRD <10^-2^ in the DPR group had a more frequent recurrence rate than those in the DGR group (*P*<0.05). For patients at LR, there was no difference in the relationship between early treatment response and recurrence between the two groups.

**Table 4:** Relationship between early treatment response and relapse in two groups.

GROUP	DGR	*P*-value	DPR	*P*-value	*P*-value
LR					
D_19_MRD		<0.05		0.89	
<10^-4^	79/1364 (5.8%)		16/178 (9%)		0.098
10^-3^–10^-4^	42/488 (8.6%)		5/55 (10.9%)		0.53
10^-2^–10^-3^	66/456 (14.5%)		11/109 (10.1%)		0.148
D_46_MRD		<0.05		1	
<10^-4^	168/2228 (7.5%)		32/331 (9.7%)		0.11
10^-4^–10^-2^	19/80 (23.8%)		1/11 (9.1%)		0.445
IR/HR					
D_19_MRD		<0.05		0.013	
<10^-4^	69/702 (9.8%)		36/223 (16.1%)		0.008
10^-3^–10^-4^	26/155 (16.7%)		14/70 (20%)		0.707
10^-2^–10^-3^	66/293 (22.5%)		28/132 (21.2%)		0.707
>10^-2^	73/339 (21.5%)		93/336 (27.7%)		0.197
D_46_MRD		<0.05		<0.05	
<10^-4^	167/1318 (12.7%)		113/598 (18.9%)		<0.05
10^-4^–10^-2^	81/322 (25.2%)		58/163 (35.6%)		<0.05

LR, low risk; HR, high risk, IR, intermediate risk; D_19_MRD, minimal residual disease on day 19 of induction; D_46_MRD, minimal residual disease on day 46 of induction.

### Relationship between dexamethasone treatment response and recurrence

The overall recurrence rate in the DGR group was lower than that in the DPR group (11% vs. 18.6%). However, in the LR and HR patients, dexamethasone response was not associated with recurrence (*P*>0.1), while the patients at IR in the DGR group had a lower recurrence rate than that of those in the DPR group (*P*<0.05; **Table 5**).

**Table 5:** Relationship between dexamethasone treatment response and recurrence.

Group	DGR	DPR	*P*-value
LR	187/2308(8.1%)	32/342(9.36%)	0.432
IR	248/1640(15.12%)	171/761(22.47%)	<0.05
HR	7/62(11.29%)	11/48(22.92%)	0.102
L/I/HR	442/4010(11%)	214/1151(18.6%)	<0.05

DGR, dexamethasone good response; DPR, dexamethasone poor response; LR, low risk; HR, high risk, IR, intermediate risk.

### Relationship between dexamethasone response and central nervous system relapse

Of the 1,151 patients in the DPR group, 51 (4.4%) had CNS relapse, and of the 4,010 patients in the DGR group, 76 (1.9%) had CNS relapse. As reported in **Table 6**, CNS3 and CNS2 lesions at the time of initial diagnosis as well as the first intrathecal injection had no effect on the later development of CNS relapse (*P*>0.1). However, the recurrence rate of CNS relapse was significantly higher in the DPR than in the DGR group (*P*<0.05), indicating that poor response to dexamethasone was a risk factor for recurrence of CNS relapse.

**Table 6:** Relationship between central nervous system relapses and CNS3, CNS2 or the first intrathecal injection injury at the initial diagnosis.

	Central nervous system relapses		No central nervous system relapses		*P*-value
	a	b	a	b	
DGR	6	70	303	3404	0.93
DPR	9	42	127	973	0.187

a indicates CNS3, CNS2, or the first intrathecal injection injury at initial diagnosis.

b Indicates CNS1 without the First Intrathecal Injection Injury at the Initial Diagnosis.

CNS, central nervous system; DGR, dexamethasone good response; DPR, dexamethasone poor response.

### Comparison of the 6-year EFS rate between the DGR and DPR groups

**Figure 2:**
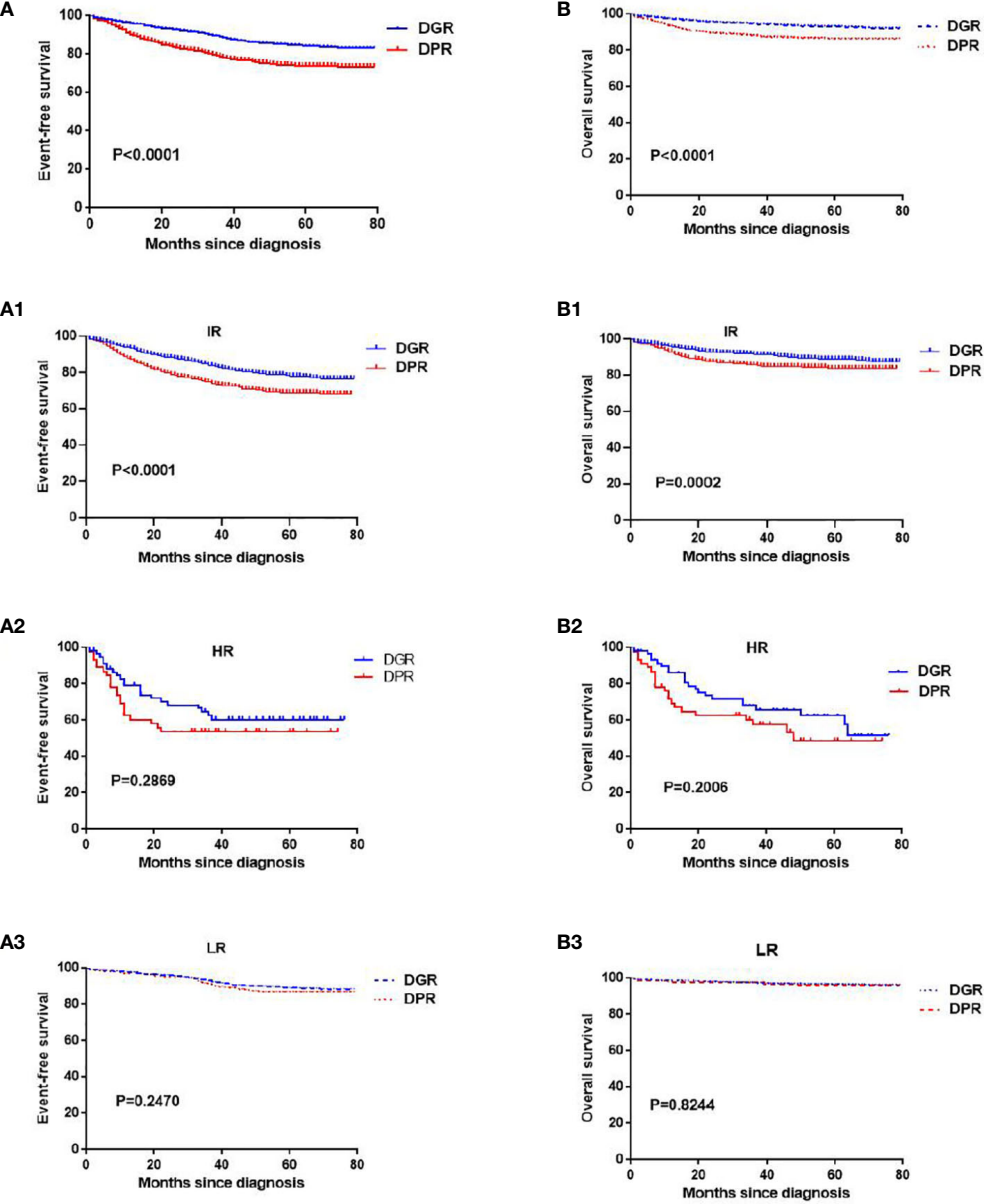
Kaplan–Meier analyses of the event-free survival and overall survival in the DGR and DPR groups. Panels (**A, A1**–**A3**) indicate the event-free survival of all patients, patients at intermediate risk, patients at high risk, and patients at low risk, respectively. Panels (**B, B1**–**B3**) indicate the overall survival of all patients, patients at intermediate risk, patients at high risk, and patients at low risk, respectively.

The DGR group compared with the DPR group had a significantly higher 6-year EFS rate (83% vs. 73%; *P*<0.05; **Figure 2A**). The 6-year EFS rate for IR children was approximately 80% in the DGR group, which was better than that in the DPR group (approximately 70%; *P*<0.05; **Figure 2A1**).

However, the 6-year EFS rates for children at LR were >85% in both groups, with no significant difference between the two groups (*P*=0.24; **Figure 2A3**), and the 6-year EFS rates for children at HR were not significantly different between the two groups (*P*=0.159; **Figure 2A2**).

### Comparison of the 6-year OS rates between the DGR and DPR groups

The DGR group compared with DPR group had a significantly higher 6-year OS survival (92% vs. 86%, *P*<0.05; **Figure 2B**). The 6-year OS rates for children at IR were significantly different between the two groups (88% vs. 83%, *P*<0.05; **Figure 2B1**). However, the 6-year OS rates for children at HR and LR were not significantly different between the two groups (*P*>0.05; **Figures 2B2, 2B3**).

## Discussion

In our study, the recurrence rate and the proportion of patients at IR were higher in the DPR than in the DGR group, which is consistent with the findings of previous trials (10, 11). Age distribution was significantly different between the DGR and DPR groups, with a significantly higher proportion of patients aged ≥10 years in the DPR than in the DGR group. The recurrence, 6-year EFS, and 6-year OS rates were not associated with dexamethasone response in patients at LR and HR, but only in those at IR, where both the EFS and OS rates at 6 years were better in the DGR than in the DPR group (*P*<0.001). Univariate analysis indicated that the male-to-female ratio in the DPR group was higher than that in the DGR group, perhaps because of the dominance of patients at IR and HR in the DPR group and the higher male-to-female ratio in the IR than that in the LR group. However, multivariate analysis showed no correlation between dexamethasone response and sex. This result is consistent with the result of the ALL-2008 protocol, in which prednisone response was not associated with sex but showed poorer response in older patients compared to younger patients (18).

Extramedullary leukemia is an indicator of poor prognosis. The NOPHO-ALL-92 and ALL-2000 trials found that compared with an initial diagnosis of leukemia with no CNS involvement, an initial diagnosis of CNS leukemia was more frequent in patients with T-ALL, hyperleukocytosis, and *BCR/ABL* fusion gene positivity (19). In our study, most of the patients with CNS involvement at the time of initial diagnosis had poor response to dexamethasone, and patients with poor response to dexamethasone had higher incidences of T-ALL, hyperleukocytosis, and *BCR/ABL* fusion gene positivity than that of patients with good response to dexamethasone. This finding is consistent with those reported in the literature.

T-ALL is an established indicator of poor prognosis and high recurrence rates. Compared with B-ALL, T-ALL is often accompanied by a high WBC count, mediastinal masses, and CNS infiltration, and patients with T-ALL have poor responses to prednisone and unsatisfactory survival and prognosis (20, 21). In our study, the proportion of patients with T-ALL was significantly higher in the DPR than in the DGR group, in line with previous findings.

However, the multivariate analysis showed no correlation between T-ALL and dexamethasone response, perhaps because patients with T-ALL were excluded, similar to patients at LR in the study.

Genetic abnormalities are important for risk stratification and treatment guidance in children with ALL. Multicenter studies have shown that patients with hyperdiploid and *ETV6-RUNX1* fusion genes have good prognosis and that the presence of the *BCR/ABL1, TCF3/PBX1, PDGFRB*, and *MLL* fusion genes often indicates poor prognosis (22). In this study, the proportion of abnormal chromosomal karyotypes was higher in the DPR than in the DGR group. Furthermore, the proportion of patients who tested positive for the *ETV6-RUNX1* fusion genes in the DGR group (21.05%) was significantly higher than that in the DPR group (11.64%), which was consistent with the findings by Zhen et al. who stated that patients positive for the *ETV6-RUNX1* fusion gene had good hormone responses (22). In addition, the high detection rates of the *BCR/ABL1* and *PDGFRB* fusion genes in the DPR group in this study were consistent with previous findings (23). Previous studies have reported that patients positive for *MLL* gene rearrangements have high WBC counts, CNS involvement, relative resistance to glucocorticoids and L-ASP, and early recurrence (24) and that the *MLL* gene is not associated with dexamethasone response (23). Most *TCF3/PBX1*-positive patients have been shown to have adverse prognostic factors, such as high WBC count and older age (25, 26). In the NPCLC-ALL-2008 trial, the complete response, prednisone response, and recurrence rates were not significantly different between *TCF3/PBX1*-positive and *TCF3/PBX1*-negative patients (27). However, the high proportion of *TCF3/PBX1*-positive patients in the DPR group in this study was not consistent with the single-center results of the NPCLC-ALL-2008 trial, which may be related to the difference in the sample size.

Many studies have shown that bone marrow MRD is the most reliable independent predictor of survival and leukemia recurrence in children with ALL and that high MRD after induction chemotherapy is closely related to leukemia recurrence (28). Currently, an MRD of 10^-4^ is used as the positive threshold for MRD worldwide, but there is no consensus on the time point for assessing MRD (29). The CCCG-ALL-2015 protocol assessed MRD on day 46 of chemotherapy and found that patients with persistently positive D_46_MRD had poor prognosis (30). Some international studies have suggested that D_19_MRD ≥10^-2^ indicates poor prognosis (31). In this study, D_19_MRD was used as the basis for assessing early treatment effects and risk adjustments; patients with D_19_MRD ≥10^-2^ were considered as being at IR; the intensity of chemotherapy should be strengthened for such patients.

Patients with a D_46_MRD ≥10^-2^ were considered to be at HR, and stem cell transplantation should be performed as soon as possible for such patients.

In this study, there was a correlation between dexamethasone response and early treatment response. The proportion of patients with D_19_MRD ≥10^-2^ or D_46_MRD ≥10^-4^ in the DPR group was significantly higher than that in the DGR group, a result that was consistent with the findings by Yu et al. who stated that DPR was a risk factor for D_19_MRD ≥10^-2^ (32). In addition, early treatment response and recurrence of leukemia were different among children with different dexamethasone responses. Early treatment response was not associated with prognosis in HR children in the DGR group, but the recurrence rates were significantly higher in children at LR with D_19_MRD ≥10^-3^ or in those at IR with D_19_MRD ≥10^-4^. Moreover, the recurrence rate for children at IR with D_19_MRD ≥10^-2^ was comparable to that for those with 10^-4^ ≤D_19_MRD <10^-3^ but lower than that for children at IR with 10^-3^ ≤D_19_MRD<10^-2^. This is probably attributed to the additional course of CAT+ consolidation therapy in the former. Intensified chemotherapy can be administered to reduce recurrence in children at LR with D_19_MRD ≥10^-3^ or at IR with D_19_MRD ≥10^-4^. The recurrence rate for patients with a D_46_MRD ≥10^-4^ was significantly higher than that for other patients, indicating that it is scientifically feasible to set MRD detection on d46 of chemotherapy. D_19_MRD, D_46_MRD, and recurrence rates were not significantly different between patients at LR and HR in the DPR group, whereas D_19_MRD ≥10^-4^ and D_46_MRD ≥10^-4^ in patients at IR suggested an increased risk of recurrence. In addition, patients with D_19_MRD ≥10^-2^ in the DPR group still had a high recurrence rate after a course of enhanced CAT+ chemotherapy, indicating that the leukemia cells of patients with poor response to dexamethasone are generally resistant to glucocorticoids and exhibit multidrug resistance (11). The dexamethasone response was especially important for judging the prognosis of patients at IR. In addition to enhancing chemotherapy, technologies, such as second-generation sequencing, should be used to identify HR factors that affect prognosis, and targeted therapy should be sought. In summary, regardless of the dexamethasone response, early treatment response was not correlated with prognosis in patients being at HR.

## Data availability statement

The raw data supporting the conclusions of this article will be made available by the authors, without undue reservation.

## Ethics statement

The studies involving human participants were reviewed and approved by IRB of Shanghai Children’s Medical Center Affliated Shanghai Jiaotong University School of Medicine. Written informed consent to participate in this study was provided by the participants’ legal guardian/next of kin.

## Author contributions

Conceptualization: SH and NW. Methodology: JinC, HC and JiaoC. Data: HC and XB. Original Draft Preparation and writing: JinC and HC. Writing – Review & Editing: SH and NW. Investigation: YC, XG, XC, HJ, XZ. Project administration: YF, LZ, XT, FZ, YW, LW, and HL. Visualization: WL, MY, HY, and AZ. All authors contributed to the article and approved the submitted version.
